# A field resource for the glioma cerebrospinal fluid proteome: Impacts of resection and location on biomarker discovery

**DOI:** 10.1093/neuonc/noae277

**Published:** 2024-12-30

**Authors:** Cecile Riviere-Cazaux, Christopher J Graser, Arthur E Warrington, Matthew D Hoplin, Katherine M Andersen, Noor Malik, Elizabeth A Palmer, Lucas P Carlstrom, Surendra Dasari, Amanda Munoz-Casabella, Samar Ikram, Keyvan Ghadimi, Benjamin T Himes, Ignacio Jusue-Torres, Jann N Sarkaria, Fredric B Meyer, Jamie J Van Gompel, Sani H Kizilbash, Ugur Sener, Franziska Michor, Jian L Campian, Ian F Parney, Terry C Burns

**Affiliations:** Department of Neurological Surgery, Mayo Clinic, Rochester, Minnesota, USA; Department of Stem Cell and Regenerative Biology, Harvard University, Cambridge, Massachusetts, USA; Department of Biostatistics, Harvard T. H. Chan School of Public Health, Boston, Massachusetts, USA; Department of Data Science, Dana-Farber Cancer Institute, Boston, Massachusetts, USA; Department of Neurological Surgery, Mayo Clinic, Rochester, Minnesota, USA; Department of Neurology, Mayo Clinic, Rochester, Minnesota, USA; Department of Neurological Surgery, Mayo Clinic, Rochester, Minnesota, USA; Department of Neurological Surgery, Mayo Clinic, Rochester, Minnesota, USA; Department of Neurological Surgery, Mayo Clinic, Rochester, Minnesota, USA; Department of Neurological Surgery, Mayo Clinic, Rochester, Minnesota, USA; Department of Neurological Surgery, The Ohio State University, Columbus, Ohio, USA; Department of Neurological Surgery, Mayo Clinic, Rochester, Minnesota, USA; Department of Quantitative Health Sciences, Mayo Clinic, Rochester, Minnesota, USA; Department of Neurological Surgery, Mayo Clinic, Rochester, Minnesota, USA; Department of Neurological Surgery, Mayo Clinic, Rochester, Minnesota, USA; Department of Neurological Surgery, Montefiore/Albert Einstein College of Medicine, Bronx, New York, USA; Department of Neurological Surgery, Montefiore/Albert Einstein College of Medicine, Bronx, New York, USA; Department of Neurological Surgery, Mayo Clinic, Rochester, Minnesota, USA; Department of Radiation Oncology, Mayo Clinic, Rochester, Minnesota, USA; Department of Neurological Surgery, Mayo Clinic, Rochester, Minnesota, USA; Department of Neurological Surgery, Mayo Clinic, Rochester, Minnesota, USA; Department of Medical Oncology, Mayo Clinic, Rochester, Minnesota, USA; Department of Neurology, Mayo Clinic, Rochester, Minnesota, USA; The Eli and Edythe L. Broad Institute of MIT and Harvard, Cambridge, Massachusetts, USA; The Ludwig Center, Harvard University, Cambridge, Massachusetts, USA; Department of Stem Cell and Regenerative Biology, Harvard University, Cambridge, Massachusetts, USA; Department of Biostatistics, Harvard T. H. Chan School of Public Health, Boston, Massachusetts, USA; Center for Cancer Evolution, Dana-Farber Cancer Institute, Boston, Massachusetts, USA; Department of Data Science, Dana-Farber Cancer Institute, Boston, Massachusetts, USA; Department of Medical Oncology, Mayo Clinic, Rochester, Minnesota, USA; Department of Neurological Surgery, Mayo Clinic, Rochester, Minnesota, USA; Department of Neurological Surgery, Mayo Clinic, Rochester, Minnesota, USA

**Keywords:** biomarker | cerebrospinal fluid | glioma | monitoring | proteomics

## Abstract

**Background:**

While serial sampling of glioma tissue is rarely performed prior to recurrence, cerebrospinal fluid (CSF) is an underutilized longitudinal source of candidate glioma biomarkers for understanding therapeutic impacts. However, the impact of key variables to consider in longitudinal CSF samples for monitoring biomarker discovery, including anatomical location and post-surgical changes, remains unknown.

**Methods:**

Aptamer-based proteomics was performed on 147 CSF samples from 74 patients; 71 of whom had grade 2–4 astrocytomas or grade 2–3 oligodendrogliomas. This included pre- versus post-resection intracranial CSF samples obtained at early (1–16 days; *n* = 20 patients) or delayed (86–153 days; *n* = 11 patients) time points for patients with glioma. Paired lumbar versus intracranial glioma CSF samples were also obtained (*n* = 14 patients).

**Results:**

Significant differences were identified in the CSF proteome between lumbar, subarachnoid, and ventricular CSF in patients with gliomas. Importantly, we found that resection had a significant, evolving longitudinal impact on the CSF proteome, with distinct sets of proteins present at different time points since resection. Our analysis of serial intracranial CSF samples suggests the early potential for disease monitoring and evaluation of pharmacodynamic impact of targeted therapies, such as bevacizumab and immunotherapies.

**Conclusions:**

The intracranial glioma CSF proteome serves as a rich and dynamic reservoir of potential biomarkers that can be used to evaluate the effects of resection and other therapies over time. All data within this study, including detailed individual clinical annotations, are shared as a resource for the neuro-oncology community to collectively address these unanswered questions and further understand glioma biology through CSF proteomics.

**Key Points**

- There are substantial differences in the glioma cerebrospinal fluid (CSF) proteome across anatomical sites.
- Resection has a significant temporal impact on the glioma CSF proteome.

Longitudinal CSF can be acquired for early pharmacodynamic evaluation of systemic therapies.

**Importance of the Study**

Improved methods for disease monitoring are needed for patients with high-grade gliomas. Toward rigorously identifying cerebrospinal fluid-based monitoring biomarkers, we sought to evaluate the impacts of key longitudinal variables on the glioma cerebrospinal fluid (CSF) proteome, namely anatomical site of acquisition and resection. Our study revealed significant differences in the glioma CSF proteome across lumbar, subarachnoid, and ventricular CSF samples. Moreover, resection had a lasting, dynamic impact on longitudinal CSF samples independent of changes in tumor burden, even months after surgery. This work not only demonstrates the feasibility of repeated CSF sampling for tracking pharmacodynamic changes but also provides valuable insights into the temporal evolution of the glioma CSF proteome post-surgery and treatment toward calibrating the discovery of monitoring biomarkers. We provide all proteomic data and clinical annotations as a resource to the neuro-oncology community to support and enhance collaborative efforts in the discovery of CSF biomarkers for patients with gliomas.

Gliomas are primary brain tumors that inevitably recur despite maximal safe surgical resection and aggressive chemoradiation.^1^ Magnetic resonance imaging (MRI) is routinely used for disease monitoring but remains subject to confounders including pseudoprogression.^2–6^ Additionally, standard sequences lack data on glioma biology. Liquid biopsies may serve as a source for monitoring and pharmacodynamic biomarker.^7–10^ While the blood-brain barrier (BBB) limits the passage of glioma-derived analytes into plasma,^11–16^ cerebrospinal fluid (CSF) is more proximal to the tumor for improved brain tumor biomarker detection,^17–19^ including cell-free DNA^9,20–24^ and 2-hydroxyglutarate for isocitrate dehydrogenase (IDH)-mutant gliomas.^25–28^ In contrast, the glioma CSF proteome remains relatively under-explored,^29–33^ particularly for longitudinal monitoring and pharmacodynamics.^34^ As a protein-deficient fluid, CSF provides improved signal to noise for biomarker discovery compared with plasma. However, the lumbar and intracranial CSF proteomes are known to be different,^35^ which may impact biomarker identification. Indeed, optimal monitoring sensitivity is obtained from tissue-contacting biofluids^36,37^ that can be obtained intra-operatively or longitudinally using CSF access devices.

To understand factors relevant to disease monitoring in the longitudinal glioma CSF proteome, we conducted aptamer-based proteomics using the SomaLogic platform.^38,39^ We focused on the impact of anatomical location and resection due to their relevance for serial CSF sampling: (1) CSF may be collected from different locations before resection versus during monitoring, such as the subarachnoid space versus the resection cavity, respectively, and (2) most glioma patients undergo resection, making the longitudinal effects of surgery, independent of changes in disease burden, critical for biomarker discovery.

To address these questions, 147 CSF samples were collected from 74 patients, including 71 with grade 2–4 astrocytomas or grade 2–3 oligodendrogliomas, spanning different CSF compartments at multiple time points, pre- and post-resection. The data set includes: (1) intracranial subarachnoid or ventricular CSF samples in primary and recurrent gliomas, (2) paired lumbar and intracranial glioma samples, and (3) longitudinal glioma CSF from CSF access devices like Ommaya reservoirs (NCT04692337)^25^ to assess the effects of resection and targeted therapies. We identified a significant impact of anatomical sampling location on the glioma CSF proteome, along with dynamic and persistent changes following resection that lasted for months. The glioma CSF proteome was highly enriched with plasma-derived proteins, indicating BBB disruption. All proteomic data and sample annotations are provided to support biomarker and biology discovery and validation within the neuro-oncology community.

## Materials and Methods

### Recruitment, CSF Collection, and Processing

Cerebrospinal fluid was collected and biobanked with informed consent via one or more Institutional Review Board-approved protocols (NCT04692337, NCT04692324, or the neuro-oncology biorepository); the details are further described in Supplemental Methods. Cerebrospinal fluid was centrifuged at 400*g* for 10 min at 4°C, aliquoted, and stored at −80°C. Clinical information was documented for each sample (“Annotations,” Supplementary Data).

### Aptamer-Based Proteomics

Aptamer-based proteomics in CSF was performed on the SomaLogic SomaScan platform^38^ to detect over 7000 proteins in liquid specimens, detailed in Supplemental Methods with their data standardization and processing methods. Values normalized by adaptive normalization by maximum likelihood (ANML) were used for all analyses in this manuscript.

### Enrichment Analysis

For intra-patient paired samples, aptamer fold-changes were calculated to generate a ranked list for each comparison (eg, pre- versus post-resection). For group comparisons, average ANML values were calculated per group, and fold-change ranking was generated. Discovery and validation cohort analyses were conducted by dividing samples chronologically (first half = discovery; second half = validation). Analyses were also stratified by tumor status (primary versus re-resected), and CSF location (subarachnoid versus ventricular), where applicable. For patients with multiple intra-operative samples, the first sample was used, assuming only one sample would typically be available during surgery, unless it was part of a known bloody/clean pair, in which case the cleaner sample was used.

Gene Set Enrichment Analysis was repurposed to determine the statistical significance of similarities between ranked protein lists. Ranked fold-change lists were created for comparisons and their enrichment for different protein libraries was evaluated. See Supplemental Methods for details. After reviewing normalized enrichment scores (NES) and false discovery rates (FDRs), we set a stringent significant cutoff of FDR < 0.001 and NES ≥ 10.

### Volcano Plots, Heatmaps, and Hierarchical Clustering

Volcano plots were generated from nonparametric tests (Wilcoxon signed-rank for paired data; Mann–Whitney *U* test for unpaired data) after confirming non-normal data distribution via the D’Agostino-Pearson test. Multiple hypothesis correction was performed using the Benjamini–Hochberg method.

To identify time point-specific proteins (Figure 3A), we evaluated the proteins that were significantly elevated only in that time point when it was compared with both of the other time points independently. To identify proteins shared between 2 time points, but not the other, we evaluated the overlap of significant proteins between those 2 time points when each was independently compared with the third time point.

**Figure 1.**
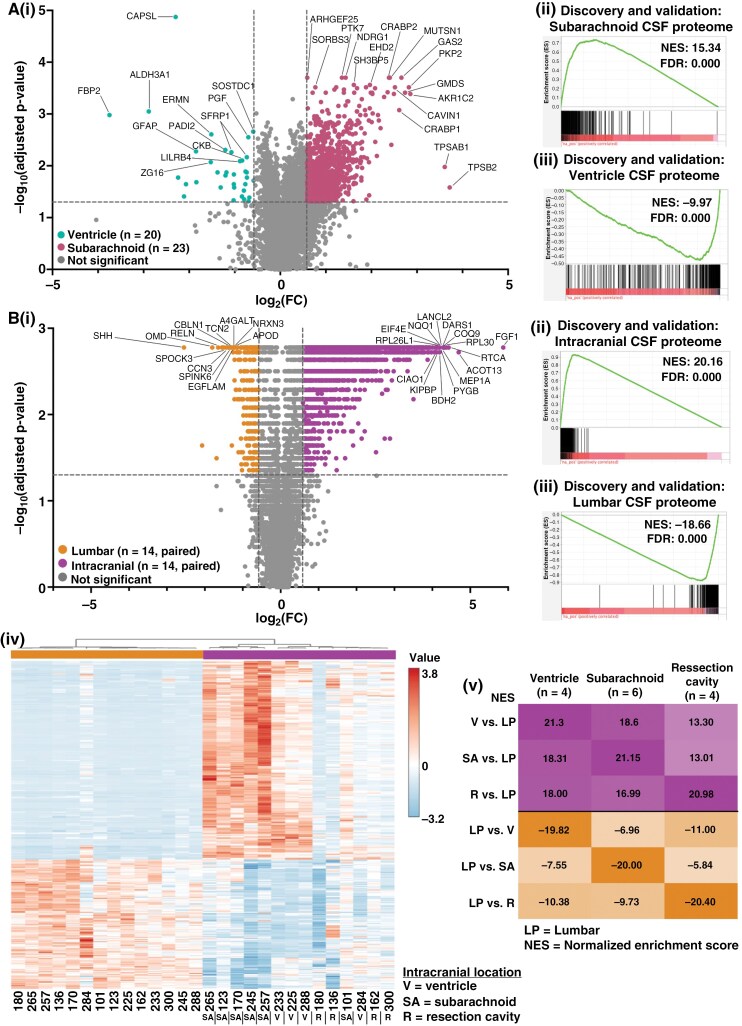
The CSF proteome differs by anatomical location. (A): (i) A volcano plot was generated by performing a series of Mann–Whitney *U* tests with Benjamini–Hochberg correction on GBM CSF samples obtained from the ventricular system (*n* = 20) versus the subarachnoid space (*n* = 23). Cutoffs were FC ≥1.5 and adjusted *P*-value ≤.05. (ii and iii) The ventricular and subarachnoid CSF samples were each split into half and categorized as discovery and validation cohorts (*n* = 10 each in discovery and validation for ventricular; *n* = 12 in discovery, and *n* = 11 in ventricular for the validation cohort), with subarachnoid versus ventricular CSF ranked fold-change lists generated for both cohorts. Enrichment analysis was performed to evaluate the enrichment of the discovery subarachnoid versus ventricular ranked fold-change list for the (ii) validation subarachnoid and (iii) validation ventricular CSF proteomes (top 350 proteins based on fold-change). Significance was set at normalized enrichment scores (NES) ≥10 and FDR <0.001. (B): (i) A volcano plot was generated by performing a series of Wilcoxon signed-rank tests with Benjamini–Hochberg correction on paired intracranial and lumbar CSF samples from patients with gliomas (*n* = 14). (ii and iii) The lumbar and intracranial pairs were split in half into discovery and validation cohorts (*n* = 7 pairs in each group), with paired intracranial versus lumbar ranked fold-change lists generated for both cohorts. Enrichment analysis was performed to evaluate the enrichment of the discovery intracranial versus lumbar ranked fold-change list for the (ii) validation intracranial and (ii) validation lumbar CSF proteomes. (iv) Hierarchical clustering was performed on the paired lumbar and intracranial CSF samples using the top 5% (350 proteins) of proteins via *t*-test. (v) Ranked average fold-change lists were generated using the paired intracranial versus lumbar patient samples based on anatomical location (ventricle, subarachnoid, or resection cavity). Enrichment analysis was performed to evaluate the enrichment of each ranked list for proteomic libraries consisting of the top 5% (350 proteins) of proteins from the average paired intracranial versus LP lists. All FDR = 0.000. Significance was set at NES ≥ 10 and FDR < 0.001.

**Figure 2.**
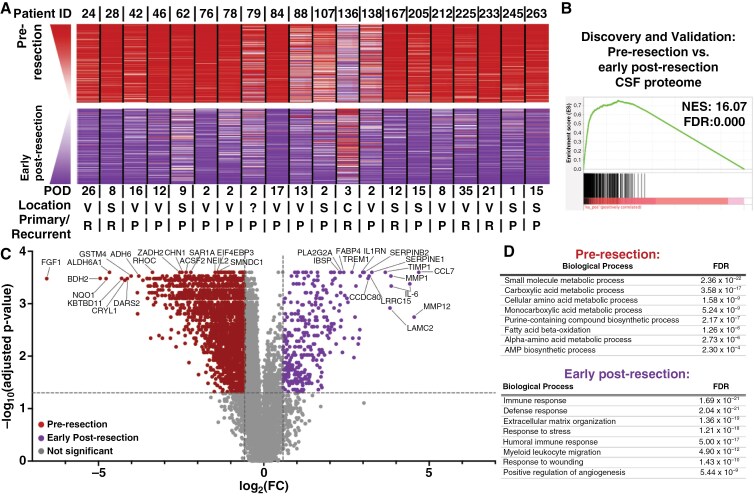
Glioma resection impacts the CSF proteome early after surgery. (A) Ranked fold-change lists were generated from each patient’s paired pre- versus post-early resection (POD ≤ 35) CSF samples (*n* = 20). Ranked protein order is shown as a heatmap from 1 (higher pre-resection; top) to 7011 (higher early post-resection; bottom). The average rank across all 20 patients was used to list proteins. The top and bottom 200 proteins are shown. (B) The 20 pre- versus early post-resection CSF pairs were split into half and categorized as discovery and validation cohorts based on sequential order. Enrichment analysis of the discovery post- versus pre-early resection ranked fold-change list was performed for the validation pre-resection CSF proteome. (C) A volcano plot was generated by performing a series of Wilcoxon signed-rank tests with Benjamini–Hochberg correction on the paired pre- versus early post-resection CSF samples (*n* = 20). Cutoffs were FC ≥ 1.5 and adjusted *P*-value ≤ .05. (D) Functional protein network analysis was performed via STRING.db on the top 200 proteins significantly elevated prior to versus early after resection based on FC in Figure 2C. Gene ontology (GO) processes and their FDRs are reported. Significance was set at NES ≥ 10 and FDR < 0.001 for all enrichment analyses.

**Figure 3.**
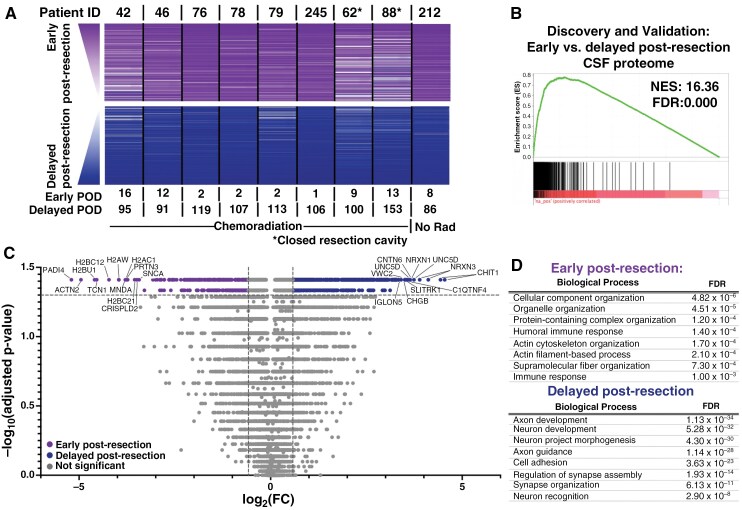
There is an evolving signature of resection between the early and delayed time points independent of chemoradiation. (A) Similar analyses to Figure 2A were performed for the paired early versus delayed post-resection CSF samples (*n* = 9 pairs; Figure 2). (B) Discovery cohort had 5 pairs of samples while the validation cohort had 4 pairs, based on sequential patient number. Significance was set at NES ≥ 10 and FDR < 0.001 for all enrichment analyses. (C) A volcano plot was generated by performing a series of Wilcoxon signed-rank tests with Benjamini–Hochberg correction on the paired early- versus-delayed post-resection CSF samples (*n* = 9). Cutoffs were FC ≥ 1.5 and adjusted *P*-value ≤ .05. (D) Functional protein network analysis was performed via STRING.db on the top 200 proteins significantly elevated at the early versus delayed time point after resection based on FC in Figure 3C. Gene ontology (GO) processes and their FDRs are reported. Significance was set at NES ≥ 10 and FDR < 0.001 for all enrichment analyses.

STRING.db was used for functional network evaluation using the top 200 proteins from fold-changes or the time point-specific proteins (Figure 3). MetaboAnalyst 6.0 was utilized for hierarchical clustering (Euclidean distance measure; Ward’s method for clustering; analysis of variance (ANOVA)/*t*-tests). Graphs were generated using GraphPad PRISM 10.0.1.

## Results

### Impact of Anatomical Location on the Intracranial CSF Proteome

Intracranial CSF can be collected from various sites, including the ventricular system and subarachnoid space. To assess the impact of sampling location on the proteome, we analyzed glioblastoma (GBM) patient samples, comparing CSF from the ventricular system versus the subarachnoid space (*n* = 20 versus 23 patients, respectively) using Mann–Whitney *U* tests with Benjamini–Hochberg correction for multiple comparisons. We identified 1033 aptamers with higher signals in subarachnoid CSF and 35 with elevated signals in ventricular CSF (fold change (FC) ≥ 1.5, adjusted *P*-value ≤.05, Figure 1Ai; Supplementary Table 1; the full list is available in Supplementary Table 2). Calcyphosine-like (CAPSL) was among the most significant ventricular-associated proteins, consistent with its known localization in ependymal cells.^40^ Functional network analysis revealed enrichment for metabolism-related pathways in subarachnoid CSF, while ventricular CSF was enriched for developmental and immune pathways (Supplementary Table 3).

To validate these findings, 10 ventricular and 12 subarachnoid GBM patient CSF samples were assigned to a discovery cohort, with the remaining patients (*n* = 10 ventricular, 11 subarachnoid) used for validation. Within each cohort, subarachnoid CSF was compared with ventricular CSF. The subarachnoid versus ventricular proteomic signature in the discovery cohort was then compared with the validation cohort using stringent cutoffs (FDR ≤0.001, NES ≥10). High reproducibility was observed between cohorts for both subarachnoid (FDR = 0.000; Figure 1Aii) and ventricular CSF proteomes (FDR = 0.000; Figure 1Aiii), confirming significant differences between these locations. Notably, subarachnoid CSF is typically collected just before resection after dural opening, whereas ventricular CSF is sampled later following tissue disruption. However, visual inspection indicated similar levels of contamination between groups (Supplementary Data, “Annotations”), suggesting that the observed differences were not due to sample contamination. Analysis of ventricular samples from VP shunts, obtained without significant tissue disruption, showed a protein signature similar to ventricular CSF collected during resection (Supplementary Figure 1), indicating that subarachnoid versus ventricular GBM CSF differences were not solely due to the timing of CSF acquisition.

### Lumbar versus Intracranial CSF Proteomes

Having identified proteomic differences based on intracranial location, we hypothesized that lumbar CSF would also differ significantly from intracranial CSF. To test this, we collected paired lumbar and intracranial CSF samples intra-operatively from 14 patients under anesthesia before tumor resection. Wilcoxon signed-rank tests with Benjamini–Hochberg correction revealed 1613 aptamers with higher signals in intracranial CSF and 271 with higher signals in lumbar CSF (FC >1.5, adjusted *P*-value <.05; Figure 1Bi; Supplementary Table 1; the full list is available in Supplementary Table 4). Intracranial CSF was enriched for metabolism-related pathways, while lumbar CSF showed enrichment for neuronal development pathways (Supplementary Table 3), consistent with elevated sonic hedgehog protein (SHH), a known craniocaudal patterning protein.^41^

Discovery and validation analyses were performed using the first 7 patients for discovery and the remaining 7 for validation. Reproducible lumbar versus intracranial CSF proteomic signatures were confirmed by enrichment analysis and hierarchical clustering (FDR = 0.000; Figure 1Bii and iii), which clearly separated paired lumbar and intracranial samples by location (Figure 1Biv). We further examined whether the lumbar versus intracranial signature remained consistent regardless of the intracranial sampling site (ventricle, subarachnoid, or resection cavity). Enrichment analysis showed positive enrichment of intracranial proteomes relative to lumbar CSF across all intracranial locations (FDR = 0.000 for most comparisons; Figure 1Bv). Thus, the intracranial CSF proteome, regardless of the sampling site, was distinct from the lumbar proteome.

### Longitudinal Impacts of Resection on the CSF Proteome

During the patient’s disease course, the largest decrease in disease burden typically occurs via surgical resection. To determine the impact of surgical resection on the CSF proteome, paired pre- and post-resection intracranial CSF samples were acquired for 20 patients early in the patient’s postoperative course (median = 10.5 days, range = 1–35). Ranked fold-change lists were created to compare each patient’s pre-resection with their post-resection sample, revealing proteomic similarities in the pre- versus post-resection signature across patients (Figure 2A). Notably, this consistent proteomic signature could be observed even in patients where post-resection CSF was obtained 3–5 weeks after resection when compared with within the first few days after surgery. To evaluate the reproducibility of these findings, the first and second 10 consecutive patients were utilized as discovery/validation cohorts, and pre- versus early post-resection CSF samples were compared within each cohort. This revealed strong reproducibility of the differences between pre- versus early post-resection CSF samples across cohorts (FDR = 0.000; Figure 2B and Supplementary Figure 2A). Given the impact of anatomical location (Figure 1), we also asked whether the impact of resection could be observed independent of whether pre-resection CSF was obtained from the ventricular system (*n* = 11 patients) or subarachnoid space (*n* = 7 patients); 2 samples were excluded from analysis as the site of origin was unknown in one patient and another originated from a resection cavity. Within the ventricular and subarachnoid CSF groups, the pre- versus early post-resection CSF samples were compared with one another. Then, the ventricular pre- versus early post-resection signature was compared with that of the subarachnoid group, revealing significant overlap indicative of a conserved impact of resection on the CSF proteome regardless of the site of origin (FDR = 0.000; Supplementary Figure 2Bi and ii). The same was also found when samples were divided based on primary (*n* = 14 patients) versus recurrence (*n* = 6 patients) status (FDR = 0.000; Supplementary Figure 2Ci and ii).

Wilcoxon signed-rank tests with Benjamini–Hochberg correction comparing the paired pre- versus post-resection samples (*n* = 20 patients) demonstrated that the signal for 1872 aptamers was significantly higher for CSF prior to resection, while the signal increased for 326 aptamers early after resection (adjusted *P* ≤ .05; FC ≥ 1.5; Figure 2C; Supplementary Table 1; the full list is available in Supplementary Table 5). Functional protein network analysis of the top 200 of the 1872 pre-resection aptamers revealed enriched gene ontology (GO) biological process terms for metabolism-related pathways, including small molecule metabolic processes (FDR = 2.36 × 10^−22^) and fatty acid beta-oxidation (1.26 × 10^−6^) (Figure 2D). Gene ontology networks enriched in the 326 post-resection proteins included immune responses (1.69 × 10^−21^), extracellular matrix reorganization (1.36 × 10^−19^), and response to wounding (1.43 × 10^−10^), consistent with recent injury from surgical resection (Figure 2D).

We recently demonstrated that the extracellular glioma metabolome is enriched for plasma-associated metabolites, consistent with relative BBB disruption in contrast-enhancing regions.^42^ As resection should decrease the abundance of glioma-associated proteins, some of which could be due to BBB disruption, we asked whether resection could alter the relative enrichment of the CSF proteome for plasma-derived proteins. To do so, we compared the pre- versus early post-resection proteome to the ranked list of plasma-derived proteins, generated from paired bloody versus clean CSF samples from 7 patients (Supplementary Table 6). As expected, plasma-derived proteins were enriched for blood coagulation and immune system-related processes, as well as cellular organization processes (Supplementary Table 3). Early post-resection CSF is often more visibly contaminated than pre-resection CSF (Supplementary Data, “Annotations”) due to post-surgical debris from recent tissue disruption. Nevertheless, plasma-associated aptamers were strongly enriched in glioma CSF at the time of maximal tumor burden when compared with early post-resection CSF (FDR = 0.000; Supplementary Figure 2Di), regardless of the subarachnoid versus ventricular origin (Supplementary Figure 2Dii and iii), primary versus recurrent status of the pre-resection sample (Supplementary Figure 2Div and v), and when analyses included only post-resection samples that were just as or more visibly contaminated than the pre-resection sample (*n* = 11/20 samples; Supplementary Figure 2Dvi). Overall, these findings aligned with our recent findings in the glioma extracellular metabolome wherein BBB-disrupted (contrast-enhancing) portions of the tumor were enriched for plasma-derived metabolites.^42^ This finding suggests that certain proteins in the CSF glioma proteome may be impacted by plasma-derived proteins that cross the tumor-disrupted BBB.

Radiation with concurrent temozolomide (TMZ) is standard of care after maximal safe resection for most patients with high-grade gliomas.^43^ We initially sought to characterize the impact of chemoradiation on the CSF proteome after resection in patients with primary gliomas. Evaluation of 8 patients’ pre- versus post-chemoradiation CSF samples seemed to indicate a proteomic difference between samples obtained prior to versus after radiation. However, paired samples from one patient who did not receive chemoradiation revealed nearly identical proteomic changes during this time frame (Supplementary Figure 3A). We therefore hypothesized that the CSF proteomic changes occurring between these early (<35 days) versus late (>70 days) time points were more strongly attributable to the ongoing evolution of dynamic postoperative changes, than chemoradiation (Figure 3A). Of note, evaluation of early and delayed time points was limited to patients with primary gliomas to minimize heterogeneity that could be induced from various times since prior resection and/or systemic treatments.

As with the pre- versus early post-resection analyses, the first 5 and second 4 patients were split into discovery and validation cohorts, respectively, and early versus delayed post-resection time points were compared within each cohort. Enrichment analysis demonstrated a reproducible impact of delayed post-resection changes on the CSF proteome when compared with the early post-resection time point (FDR = 0.000; Figure 3B; Supplementary Figure 3B). Across the 9 patients’ paired early versus delayed post-resection samples, an elevated signal was detected for 268 aptamers in the early post-resection setting while 443 aptamers had an increased signal at the delayed time point (Figure 3C; Supplementary Table 1; the full list is available in Supplementary Table 7). Pathways related to cellular component organization (FDR = 4.82 × 10^−6^), organelle organization (4.51 × 10^−5^), and immune responses (1.40 × 10^−4^) were more abundant in the early post-resection setting (Figure 3D). At the delayed time point, GO processes were enriched for neuronal-/synaptic-related pathways, including axon development (1.13 × 10^−34^), regulation of synapse assembly (1.93 × 10^−14^), and neuron recognition (2.90 × 10^−8^) (Figure 3D). Moreover, enrichment for plasma-derived proteins decreased by the delayed post-resection time point (Supplementary Figure 3C). Overall, these data suggested an increased abundance of neuronal-associated proteins as post-resection changes evolve over time.

**Figure 4.**
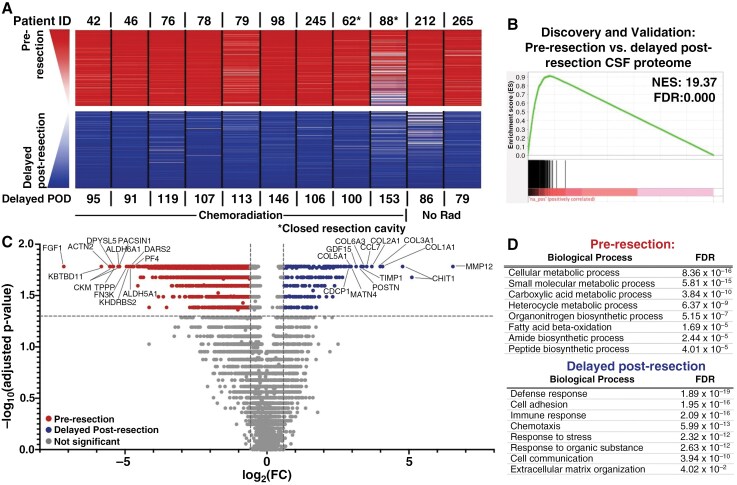
There is a conserved signature of resection between the baseline and delayed post-resection time point. (A) Similar analyses to Figures 2A and 3A were again performed using the pre-resection versus delayed post-resection CSF samples (*n* = 11 pairs). (B) Discovery cohort had 6 pairs of samples, while the validation cohort had 5 pairs, based on sequential patient order. Significance was set at NES ≥ 10 and FDR < 0.001 for all enrichment analyses. (C) A volcano plot was generated by performing a series of Wilcoxon signed-rank tests with Benjamini–Hochberg correction on the paired pre- versus-delayed post-resection CSF samples (*n* = 11). Cutoffs were FC ≥ 1.5 and adjusted *P*-value ≤ .05. (D) Functional protein network analysis was performed via STRING.db on the top 200 proteins significantly elevated at the pre-resection versus delayed post-resection time points based on FC in Figure 4C. Gene ontology (GO) processes and their FDRs are reported. Significance was set at NES ≥ 10 and FDR < 0.001 for all enrichment analyses.

Having compared pre-resection to early post-resection CSF, we then asked how the former would compare with the delayed post-resection time point. Comparing paired pre-resection versus delayed post-resection CSF samples from 11 patients resulted in similar findings (Figure 4A and B) from the pre-resection versus early post-resection comparison (Figure 2A and B; Supplementary Table 8). These findings included a highly reproducible signature across discovery/validation cohorts (*n* = 6 patients in the discovery cohort, *n* = 5 patients in the validation cohort; FDR = 0.000; Figure 4B; Supplementary Figure 4A). Gene ontology processes were enriched for metabolism-associated pathways prior to resection and for immune/stress responses at the delayed post-resection point (Figure 4C and D). Likewise, pre-resection CSF was more strongly enriched for plasma-derived proteins than the delayed post-resection time point (FDR = 0.000; Supplementary Figure 4B). In summary, the impacts of resection could be identified in the CSF proteome at both early and delayed time points, although neuronal-associated proteins became more abundant with more time since resection.

### Evolution of Post-Resection CSF Proteomic Changes

**Figure 5.**
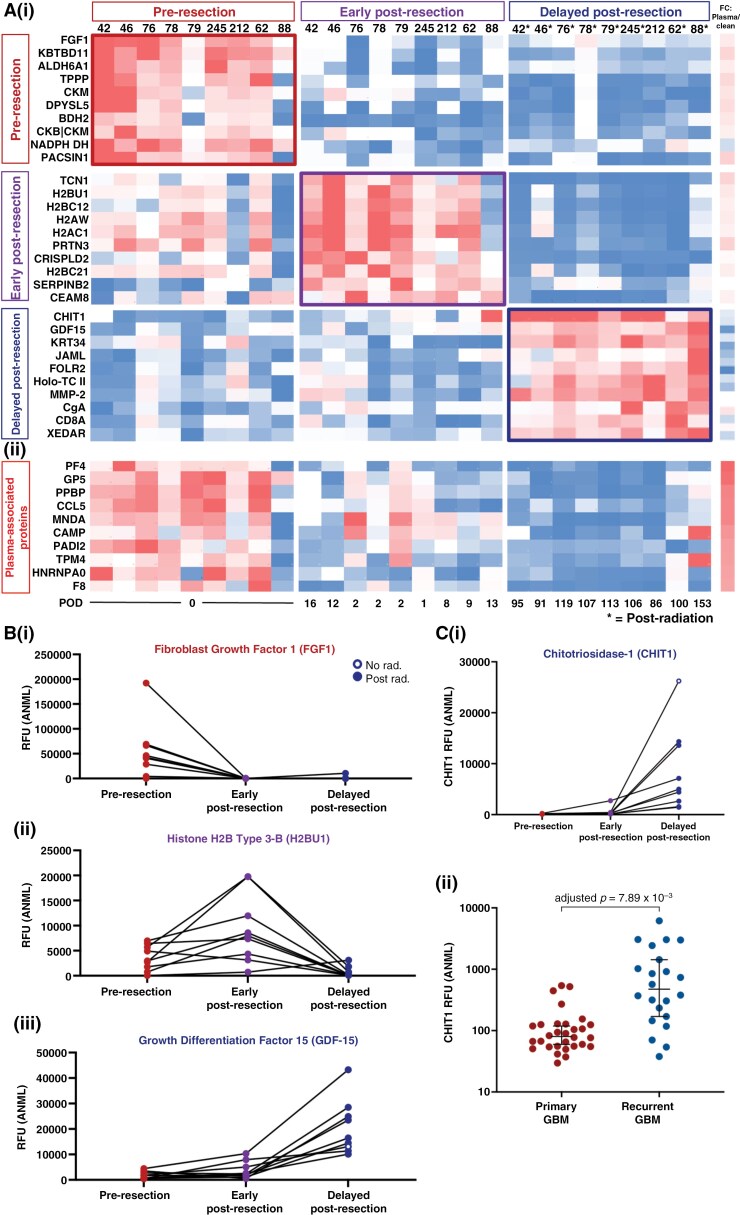
The impact of resection evolves over time and is detectable at recurrence. (A): (i) The abundance of the top 10 proteins unique to each time point is shown for 9 patients with gliomas who had CSF samples acquired intra-operatively, early post-resection (POD ≤ 35), and delayed post-resection (POD ≥ 70). Proteins unique to each time point were found by identifying the proteins that were significantly elevated (FC ≥ 1.5, adjusted *P* ≤ .05) at that time point when compared to both of the other time points. The fold-change in bloody versus clean CSF samples (*n* = 7 pairs) is also shown. (ii) The abundance of the top 10 plasma-associated proteins (based on paired bloody versus clean CSF samples) is shown for these patients’ samples. (B) The abundance at each time point for each patient is shown for (i) fibroblast growth factor 1 (FGF1), (ii) histone 2B Type 3-B (H2BU1), and (iii) growth differentiation factor-15 (GDF-15), which were in the top 10 proteins at the pre-resection, early post-resection, and delayed post-resection time points, respectively. (C) (I and ii) Chitotriosidase-1 (CHIT1) was evaluated in the pre-resection, early post-resection, and delayed post-resection time points, as well as in primary (*n* = 31) versus recurrent (*n* = 21) GBM samples acquired intra-operatively.

After comparing 2 of the 3 time points to one another, we then sought to integrate these findings to evaluate the dynamic evolution of the CSF proteome. To do so, we identified proteins that were more abundant at one time point when compared with both of the other time points (Figure 5Ai; Supplementary Table 9). These analyses identified a subset of proteins that (1) were elevated pre-resection and remained low post-resection (row 1 in Figure 5Ai; example: fibroblast growth factor 1, Figure 5Bi), (2) increased transiently in the early post-resection setting (row 2; example: histone H2B Type 3-B, H2BU1, Figure 5Bii), (3) increased early post-resection and remained elevated by the delayed post-resection time point, and (4) increased most significantly in a delayed fashion post-resection (row 3; example, growth differentiation factor-15 [GDF-15], Figure 5Biii) (Supplementary Table 9). Additionally, evaluation of the top 10 plasma-associated proteins demonstrated decreased abundance over time (Figure 5Aii), consistent with decreasing enrichment for the plasma-associated signature from pre-resection to delayed post-resection CSF. Distinct differences were noted in the functional protein network enrichments at each time point. Pre-resection proteins were enriched for metabolism-related processes and the early and delayed post-resection for stimuli response/immune processes pathways (Supplementary Table 3). Each time point was also compared with an external glioma versus normal brain tissue dataset (Supplementary Tables 10A–D). Of the 800 CSF proteins elevated pre-resection, 104 were higher in glioma than in normal brain tissue, with 76 potentially overlapping plasma-derived proteins.

Moreover, we also investigated whether proteins that increased over time after resection would remain elevated at the time of resection for recurrent glioma. To do so, we compared the unpaired proteome of primary versus recurrent GBM (*n* = 31 versus 21 patients, respectively), which identified 18 aptamers with significantly increased signal in the recurrent subgroup (FC ≥1.5, adjusted *P*-value ≤.05) (Supplementary Table 1; the full list is available in Supplementary Table 11). Of these aptamers, 2 were significantly elevated in delayed post-resection samples when compared with pre-resection samples: chitotriosidase-1 (CHIT1) and macrophage metalloelastase (MMP-12) (Supplementary Table 12). Chitotriosidase-1, a protein secreted by activated macrophages/microglia^44^ and associated with senescence,^45^ was the protein that most significantly increased after resection (Figure 5Ci) and was the second most abundant protein in recurrent GBM when compared with primary tumors (Figure 5Cii). Although there was a low number of significantly differentially enriched proteins by recurrence after *P*-value adjustment for multiple hypothesis testing, a small subset of post-resection inflammatory components may still be detectable by the time of recurrence. In summary, the CSF proteome of gliomas dynamically evolves over time with resection.

### The Dynamic CSF Proteome During Therapy

**Figure 6.**
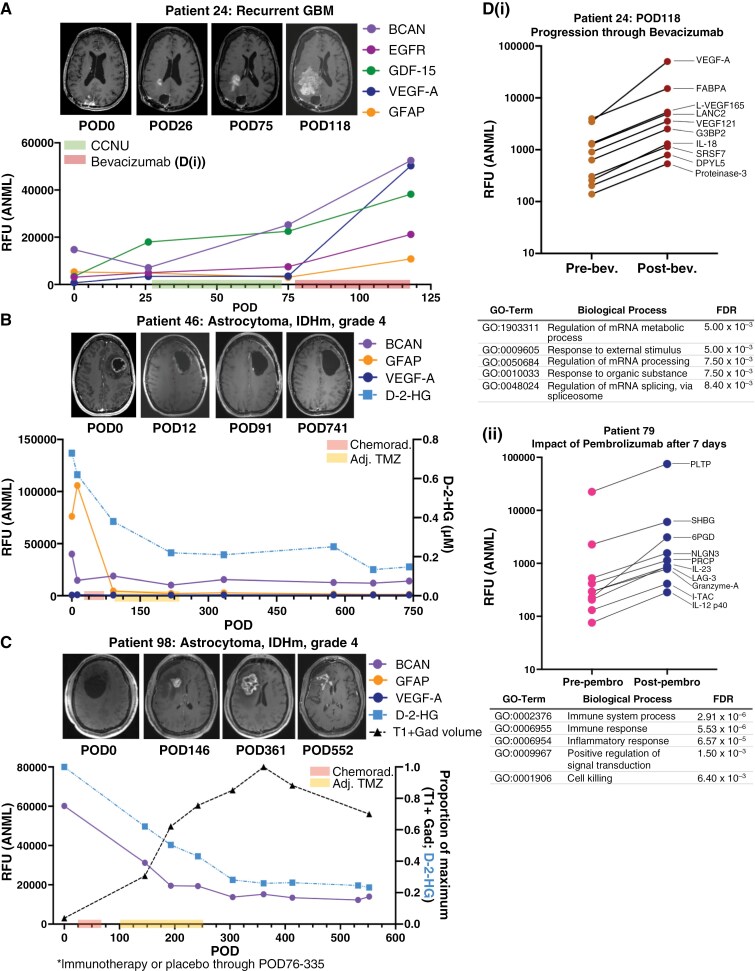
Intracranial CSF can be acquired longitudinally for evaluation of candidate monitoring biomarkers and pharmacodynamic impact of therapies. (A) Brevican (BCA), epidermal growth factor receptor (EGFR), growth differentiation factor-15 (GDF-15), vascular endothelial growth factor-A (VEGF-A), and glial fibrillary acidic protein (GFAP) were evaluated in intracranial CSF from Patient 24, who had a recurrent GBM with known EGFR amplification. The first box on the *x*-axis = days on CCNU; the second box on the *x*-axis = days on bevacizumab. (B) The normalized RFUs of BCAN, GFAP, and VEGF-A were evaluated over time in longitudinal intracranial CSF obtained from a patient with an astrocytoma, IDH-mutant, grade 4 via an Ommaya reservoir. d-2-Hydroxyglutarate (d-2-HG) was also evaluated at each time point. The first box on the *x*-axis = days during which patient underwent chemoradiation; the second box on the *x*-axis = days on adjuvant temozolomide (adj. TMZ). (C) BCAN, GFAP, and VEGF-A were evaluated in longitudinal intracranial CSF from a patient with an astrocytoma, IDH-mutant, grade 4, as well as d-2-HG. Additionally, the T1-post-gadolinium–positive (T1 + Gad) volume was calculated from MRIs obtained at each time point. The first box on the *x*-axis = days during which patient underwent chemoradiation; the second box on the *x*-axis = days on adjuvant temozolomide (adj. TMZ). (D) The top 10 proteins based on fold-change are shown for post- versus pre-treatment with (i) bevacizumab in Patient 24 from Figure 4A, as well as (ii) post- versus pre-pembrolizumab in Patient 79, who had a GBM with a hypermutated phenotype.

Beyond imaging, few methods exist to monitor glioma disease burden. We hypothesized that proteins decreasing after resection could serve as an initial set to identify candidate monitoring biomarkers. To test this, we evaluated the relative abundance of the top 25% of proteins decreasing post-resection (Figure 1A) in 3 patients with grade 4 gliomas exhibiting distinct disease courses. Patient 24, a female in her 50s, progressed through multiple treatments after resection of a recurrent GBM (Figure 6A). Patient 46, a female in her 40s, had stable disease following resection and chemoradiation of an IDH-mutant grade 4 astrocytoma (Figure 6B). Patient 98, a male in his 40s, exhibited pseudoprogression after resection and chemoradiation of an IDH-mutant grade 4 astrocytoma (Figure 6C).

We first evaluated Patient 24 as she underwent resection and had 2 definitive radiographic progressions. For monitoring, we reasoned that a biomarker should increase with true disease progression. Of the top 25% proteins, brevican (BCAN) emerged as the strongest candidate in Patient 24, increasing by 3.58× and 2.08× with progression through lomustine and bevacizumab, respectively (Figure 6A). Other proteins did not increase consistently at each progression point (Supplementary Figure 5Ai). Vascular endothelial growth factor-A (VEGF-A) and glial fibrillary acidic protein (GFAP) were evaluated a priori due to BBB disruption in HGGs^46,47^ and bevacizumab treatment, and GFAP being a proposed GBM biomarker.^48–50^ Glial fibrillary acidic protein increased (14.45×) during progression through bevacizumab, consistent with radiographic resistance to the anti-VEGF-A antibody (Figure 6A). Glial fibrillary acidic protein was among the top proteins elevated during progression through bevacizumab (3.59×) but did not increase with lomustine (1.03× and 0.63×, respectively). Epidermal growth factor receptor (EGFR) and GDF-15, a marker of p53 activity,^51^ were also assessed a priori due to Patient 24’s EGFR-amplified, p53-wild–type GBM. Both changed nominally during lomustine progression but increased 2.83× and 1.70×, respectively, during bevacizumab progression.

Next, we evaluated BCAN, GFAP, and VEGF-A in Patient 46, who had stable IDH-mutant grade 4 astrocytoma post-resection and chemoradiation. Epidermal growth factor receptor and GDF-15 were excluded due to their irrelevance to her tumor genomics. Brevican decreased to 0.61× by the end of adjuvant temozolomide at POD334 (Figure 6B), aligning with decreasing d-2-hydroxyglutarate (d-2-HG), an IDH-mutant oncometabolite. Glial fibrillary acidic protein increased 1.39× with resection and decreased to 0.96× after adjuvant temozolomide, while VEGF-A remained low since baseline. All other proteins, including those decreasing post-resection (Supplementary Figure 5Aii), remained unchanged, consistent with stable imaging. Similar protein patterns were observed in a patient with a stable GBM (Supplementary Figure 5Aiii).

In Patient 98, BCAN and GFAP signals decreased to 0.23× and 0.10× of baseline, respectively, by POD305 after standard chemoradiation with adjuvant temozolomide (Figure 6C), correlating with decreased d-2-HG. In contrast, VEGF-A increased 12.67× between post-chemoradiation and intra-operative baseline, as did several proteins elevated pre-resection that stabilized or began decreasing (Supplementary Figure 5Aiv). Despite declining BCAN, GFAP, and VEGF-A, T1-gadolinium–positive tumor volume increased until POD552, likely indicating pseudoprogression.

We also explored the feasibility of detecting pharmacodynamic signals in CSF during systemic treatment. Protein network enrichment of Patient 24’s CSF during progression through lomustine (or, chloroethylnitrosourea, CCNU) (POD26-75) revealed neuronal-related enrichment pathways similar to the early versus delayed post-resection signature, suggesting minimal pharmacodynamic impact of CCNU (Supplementary Figure 5B and Ci). Conversely, several aptamers increased during progression through bevacizumab (POD75-118), including VEGF-A (14.45×) and its isoforms, VEGF165 and VEGF121 (Figure 6Di). Gene ontology terms for bevacizumab progression included mRNA metabolic processes (5.00 × 10^−3^), response to organic substance (7.50 × 10^−3^), and regulation of mRNA splicing (8.40 × 10^−3^). Increases in these proteins contrasted with their decrease seen in other patients’ post-surgical samples (Supplementary Figure 5Cii), indicating a distinct PD impact of bevacizumab in CSF.

In another patient (Patient 79), CSF was collected before and 7 days post-pembrolizumab for primary GBM with a hypermutant phenotype (POD28-36). We observed increases in interleukins and lymphocyte activation gene-3 (LAG-3), an alternate T-cell immune checkpoint to PD153 blocked by pembrolizumab (Figure 6Dii). STRING analysis indicated increased immune system processes (FDR = 2.91 × 10^−6^) and cell-killing pathways (6.40 × 10^−3^). Again, these increases contrasted with their longitudinal decrease in other patients (Supplementary Figure 5Ciii), suggesting a potential pharmacodynamic impact of pembrolizumab in CSF.

Overall, these findings suggest that the CSF proteome may reflect biological changes during disease evolution and treatment. These example patients are solely meant as proof-of-principle for the potential of identifying CSF monitoring biomarkers. Further validation is needed to assess the reproducibility of these patterns across patients and to evaluate the performance of alternative approaches, such as individualized protein signatures based on each patient’s baseline proteome, for disease monitoring.

## Discussion

To investigate how anatomical location and resection affect the longitudinal glioma CSF proteome, 147 intracranial or lumbar CSF samples were collected from 74 patients; 71 of whom had grade 2–4 astrocytomas or grade 2–3 oligodendrogliomas. Our results demonstrate: (1) a significant impact of anatomical location on the glioma CSF proteome, particularly between subarachnoid versus ventricular and lumbar versus intracranial CSF; (2) intracranial resection-induced CSF proteomic alterations that evolve over time; (3) plasma-associated protein enrichment in pre-resection CSF that diminishes longitudinally post-resection; and (4) the feasibility of longitudinal intracranial CSF collection for biomarker hypothesis generation. While many questions remain, our data suggest that the intracranial glioma CSF proteome is a dynamic and rich source of potential biomarkers for evaluating the effects of resection and systemic therapies over time. All data are provided as a resource to support efforts in the neuro-oncology community to address these questions toward identifying glioma monitoring biomarkers.

While tissue is rarely accessible for assessing treatment response after surgery,^52^ CSF can be sampled longitudinally. We observed a conserved impact of resection on the glioma CSF proteome consistent with a tissue injury-induced inflammatory state^53,54^ that persisted for months post-surgery (Figures 2–5). For example, several histones increased in the early postoperative period, potentially reflecting their role as damage-associated molecular patterns.^55^ The increased enrichment of neuronal pathways from early (<1 month) to delayed post-resection (2–5 months) suggests recovery of normal brain CSF proteomics, likely from reduced tumor burden. Decreasing plasma-associated proteins may contribute to this finding,^56^ as clean samples in bloody versus clean pairs showed similar functional pathways (Supplementary Table 3). Since hemostatic agents dissolve within 1–2 weeks and likely have a limited proteomic profile, surgical materials are unlikely to account for these resection effects. Additionally, part of the post-resection signature may reflect the influence of chemoradiation or changes in tumor biology following surgery. Longitudinal CSF sampling from patients with low-grade gliomas (Patient 212, Figure 2), who received no therapy post-resection, or from post-biopsy patients undergoing chemoradiation, will be useful to distinguish post-resection changes from those caused by chemoradiation, other therapies, or recurrence.

The pre-resection CSF proteome was significantly enriched for plasma-derived proteins, which decreased longitudinally following resection. This decline between early and delayed post-resection samples may reflect the clearing of surgical products. However, pre-resection CSF was significantly more enriched for plasma-derived proteins even when compared with post-resection CSF that was visibly just as or more contaminated (Supplementary Figure 2Dvi). A previous study found that albumin, a plasma protein, correlated with the number of differentially abundant CSF proteins.^30^ Blood-brain barrier disruption-associated CSF proteins also correlated with poorer survival in brain metastases and CNS lymphomas.^30^ Intra-operative microdialysis in gliomas demonstrated higher plasma-derived metabolite levels in regions with BBB disruption compared with intact areas of the tumor.^42^ Thus, BBB disruption may contribute to the glioma proteome. Clean intracranial CSF (*n* = 6) was significantly more enriched for plasma proteins than the paired lumbar CSF (Supplementary Figure 2E), suggesting that the tumor itself may in part be the source of plasma-derived proteins rather than contamination. A limitation of this and similar studies is the absence of absolute contamination quantification based on hemoglobin or red blood cell levels before and after centrifugation. Future CSF collections will measure and document this variable to better distinguish BBB disruption from blood. Plasma dilution curves in CSF may also aid this analysis.

Our findings indicate that the anatomical origin of CSF, whether lumbar, subarachnoid, or ventricular, significantly influences the CSF proteome. While prior studies have shown compositional differences between intracranial and lumbar CSF in patients with NPH,^35^ similar data for glioma patients have not been reported. Cranial CSF, due to its proximity to the tumor, is expected to be a richer source of glioma-associated proteins.^9,57^ Consistent with this, our paired lumbar and intracranial glioma CSF samples revealed a greater number of differentially abundant proteins in intracranial CSF (Figure 1B), although this proximity may also increase brain-associated proteins. Notably, 1143 of the 1613 aptamers with higher signal in intracranial CSF compared with lumbar CSF decreased after resection (70.9%; Supplementary Table 12), suggesting that they may be linked to the glioma, its microenvironment, or BBB disruption. Moreover, differences between subarachnoid and ventricular CSF should be considered in longitudinal studies, as variations in tumor contact, diffusion distance, and continuity with the ventricular system can affect protein abundance. For instance, proteins like CAPSL^42^ produced by ependymal cells may be more elevated in ventricular CSF. However, this may depend on the protein under evaluation. Of the 1872 aptamers with higher signals pre-resection compared with early post-resection, 696 (37.2%) were associated with subarachnoid GBM CSF, while 1168 (62.4%) showed no significant difference between ventricular and subarachnoid CSF (Supplementary Table 13).

Intra- and inter-patient glioma heterogeneity will affect biomarker discovery. Cerebrospinal fluid reflects tumor regions it contacts, as shown by new tumor mutations in early post-resection CSF cfDNA from a hypermutated GBM.^21^ Pre- versus post-resection CSF signatures may partly reflect these changes. Proteomics from biopsies near different CSF spaces could clarify this, although differences are expected as CSF contains secreted proteins. Additional CSF samples at disease progression may reveal new biomarkers, reflecting glioma evolution. Future studies should also examine correlations between tumor genomics and CSF proteomics (Supplementary Material (Note); Supplementary Figure 6; Supplementary Table 15). A limitation of this study is that several interacting variables may influence the relative abundance of different proteins in some of the non-paired comparisons (eg, subarachnoid versus ventricular; primary versus recurrent) presented here, which are not balanced between patient groups. Subarachnoid versus ventricular and primary versus recurrent CSF analyses were unpaired due to insufficient power as such pairs are infrequently obtained. Within this unpaired analysis, the effect of CSF origin may be confounded by prior resection, as subarachnoid CSF is often collected from primary tumors due to the absence of prior tissue disruption. Of the 23 patients with subarachnoid CSF GBM samples, 20 had primary tumors, whereas the 20 patients with ventricular CSF samples were more evenly split between primary and recurrent tumors (9 and 11, respectively). Discovery and validation analyses were conducted when sample size permitted to assess the reproducibility of key findings. Collecting additional paired samples, although rare, will be crucial for validating these findings. To preliminarily address these imbalances, a linear regression model controlling for these variables was evaluated (Supplementary Material (Note); Supplementary Tables 13 and 14). These findings are exploratory due to the limited number of observations in certain categories (e.g., only 3 recurrent tumors in the subarachnoid GBM group). Additional covariates, including ones not yet identified, could further refine these models. Ongoing efforts aim to expand the cohort to support more robust analyses. In the interim, all findings have been consolidated into an interactive table of the 7011 aptamers to facilitate exploration and hypothesis generation (Supplementary Table 12). Additionally, it remains unknown whether these anatomic and surgical impacts can be replicated in glioma subtypes beyond grade 2–4 astrocytomas and grade 2–3 oligodendrogliomas, which constitute the majority of diffuse malignant gliomas.

Despite the need for careful consideration of potential covariates, our data demonstrate the feasibility of detecting unique biological effects of tumor-targeted therapies in a patient’s longitudinal CSF (Figure 6D). For example, pembrolizumab in a hypermutant tumor early after resection increased the abundance of numerous cytokines and chemokines,^58^ including LAG-3, which is currently under clinical evaluation in GBM in combination with pembrolizumab, highlighting the potential to identify emerging resistance mechanisms through CSF analysis.^59^ Of note, LAG-3 increased months after surgery, indicating that resection-associated temporal CSF changes should be considered when assessing PD impact. Progression through CCNU and bevacizumab had distinct CSF proteomic profiles, including >20× upregulation of VEGF-A which may illustrate tumor microenvironment resistance, although this requires evaluation across more patients.

In summary, the data highlight the potential of longitudinal CSF proteomics for monitoring and pharmacodynamic biomarker discovery and validation. To the best of our knowledge, this is the first study to examine the effects of tumor location and resection on the CSF proteome. By sharing these previously unreported variables and annotated proteomic data, we lay the groundwork for future research requiring functional validation of identified monitoring biomarkers. While the aptamer-based panel offers broad protein analysis for discovery, validation of candidate protein biomarkers across multiple platforms will be essential to enhance robustness, including targeted analyses performed under Clinical Laboratory Improvement Amendments (CLIA) standards. Additionally, despite being frequently altered in glioma, post-translational modifications (PTMs), such as glycosylation and phosphorylation, are rarely evaluated in CSF and could yield further insights into longitudinal glioma biology. Finally, paired tissue proteomics may serve as a valuable reference for identifying candidate CSF biomarkers (Supplementary Table 10A).

## Supplementary material

Supplementary material is available online at *Neuro-Oncology* (https://academic.oup.com/neuro-oncology).

noae277_suppl_Supplementary_Figures

noae277_suppl_Supplementary_Data

noae277_suppl_Supplementary_Tables

## Data Availability

All data, including proteomic data and clinical annotations, are available as supplementary files. Any other data can be requested from the corresponding author.
