# Supplementary material for: A field resource for the glioma cerebrospinal fluid proteome: Impacts of resection and location on biomarker discovery
Source: Neuro Oncol. 2024 Dec 30;27(4):948–62. doi: 10.1093/neuonc/noae277 (PMC12083222; doi:10.1093/neuonc/noae277)
Supplement: noae277_suppl_Supplementary_Figures [file noae277_suppl_supplementary_figures.docx]

**Supplementary Materials**

**Supplementary Methods**

*Patient recruitment, informed consent, and IRB approvals*

Samples collected intra-operatively from the surgical field were banked under our neuro-oncology biorepository protocol. Any patient undergoing resection was eligible for enrollment into the biorepository protocol. Additionally, longitudinal CSF sampling was enabled in most cases by an Ommaya reservoir implanted under a research protocol for brain tumor biomarker access at the time of tumor resection (NCT04692337). For this initial study, patients with suspected or known low-grade or high-grade gliomas were recruited. Moreover, some samples were accessed via a clinically-indicated ventriculoperitoneal shunt or post-operative external ventricular drain under our CSF biomarkers study (NCT04692324). The CSF biomarkers study was also utilized to obtain lumbar CSF from lumbar punctures, either intra-operatively after anesthesia induction or in clinic.

*Ommaya placement and CSF collection*

Intra-operatively obtained samples were most often acquired from an exposed sulcus after dural opening, a prior resection cavity, and/or once the ventricle was reached, when applicable. Efforts were taken to minimize blood contamination of each sample. Relative sample contamination was annotated whenever possible by visual inspection, which included noting the relative color of the sample (prior to versus after centrifugation, whenever possible; clear, yellow, and shades of pink to red for bloodiness), as well as whether debris was present in CSF. We also noted if the sample looked frankly bloody, based on similarity to fresh blood. For patients with an Ommaya reservoir, the catheter was placed in the resection cavity at the completion of the case—most of which contacted the ventricle, except where annotated in the figures. Most Ommayas utilized had a 1.5 cm side with a flat bottom and side inlet connected to an antibiotic-impregnated ventricular catheter (Natus ® NT8501214; Codman ® Hakim Bacisteal Catheter, NS5048). Ommayas were placed such that would be minimally visible while ensuring the catheter did not cross the incision line.

*Aptamer-based proteomics*

The Somalogic SomaScan® platform utilizes protein-captured Slow Off-rate Modified Aptamers (SOMAmers ®) for aptamer-based proteomicsto sensitively and reproducibly (<5% coefficient of variance). Some of these single-stranded DNA-based SOMAmer reagents have been chemically modified and incorporated into nucleotide libraries for selection and amplification via Systematic Evolution of Ligands by EXponential enrichment (SELEX)^1^. Although originally utilized for human plasma, serum, and urine, this technology is now being deployed in CSF^2^.

Details of this method have been more extensively detailed in prior publications^3^. Briefly, 20 uL of CSF are diluted and incubated with SOMAmer reagent mixes including streptavidin (SA)-coated beads. After the beads are washed, an NHS-biotin agent is utilized to tag the proteins specifically bound by SOMAmer reagents. Ultraviolet light then cleaves the linker within the SOMAmer reagent to release the SOMAmer complex and unbound reagents into an anionic solution, causing any nonspecific interaction to dissociate and preventing reformation. Beads are then used to separate the photocleavage eluate prior to incubation with a second set of SA-coated beads which bind biotin-labeled proteins and those complexed with SOMAmers. Another washing step is utilized to remove non-specifically bound reagents. Finally, protein-bound SOMAmer reagents are released via denaturation to quantify the reagents via hybridization to custom DNA microarrays based on a cyanine-3 signal in the reagent.

*Data standardization and processing on SomaScan platform*

The 96-well plate utilized by Somalogic includes control, quality (pooled), calibrator (pooled), and buffer (no protein) samples. Raw data obtained from hybridization undergo hybridization normalization. Hybridization normalization uses twelve control SOMAmer reagents that are added to the eluate prior to scanning the microarray, without exposure to proteins. Median signal normalization is then performed using pooled calibrator replicates to normalize for within-run technical variation. Then, for each SOMAMer® reagent, a scaling factor is calculated and performed, including 1) the plate scale (median ratio; to adjust for differences in signal intensity between runs) and 2) the calibration scale (recalculated set of scale factor based on calibrators; to adjust for SOMAmer reagent assay differences between runs). A scaling factor is then applied to the microarray based on a scaling factor from the control signals. Adaptive Normalization by Maximum Likelihood (ANML) normalization is then performed. Each SOMAmer reagent has a scale factor within a dilution bin (S1, S2, S3) that is calculated based on sample values within two population standard deviations of the normal reference. This process is then repeated until convergence to increase the likelihood that the RFU for a sample is from the reference distribution.

Samples were sent across multiple batches. To evaluate for potential batch effects, a small subset of samples was sent across multiple batches. Data were provided by Somalogic for each step in their analysis pipeline. As another normalization technique, we had also performed 1) Trimmed Mean of M-values normalization^43^ (based on top and bottom 5%) and 2) median normalization based on the median value within a sample on Somalogic’s hybridization normalized data. To determine which data normalization strategy to utilize for our analyses, we evaluated the correlation of our duplicate samples across batches to one another. Overall, ANML normalized data had the strongest correlation between batches for repeat samples (R^2^= 0.94-0.97, 5 samples) and was utilized for all analyses thereafter. Proteins flagged in any batch were removed from downstream analyses, resulting in 7,011 analyzable aptamers compared to a starting point of 7,596 aptamers.

**Enrichment analyses**

To repurpose the Gene Set Enrichment Analysis tool for proteomic enrichment, we first created our own libraries derived from our data. Libraries were generated using the top 5% of proteins based on fold-changes in the comparison (350 proteins) or the plasma-derived proteins with adjusted p-values<0.1 (**Supplementary Data**, “GSEA_Libraries). The ranked lists (.rnk) were then utilized to determine where the proteins in each library fell on the ranked list. Ranked lists were based on fold-changes across comparisons of interest. The pre-rank function was utilized with classic enrichment statistic, mean-divided normalization, and 1000 permutations. Positive enrichment indicated that the library’s proteins fell toward the top of the list, suggesting an overlap in their upregulated proteomes. Negative enrichment indicated that the ranked proteome was similar to the opposite end of the ranked list.

**Supplementary Figures and Figure Legends**

**Supplementary Figure 1. The ventricular versus subarachnoid CSF signature derived from shunt placement is similar to that of the one from resection.**

**
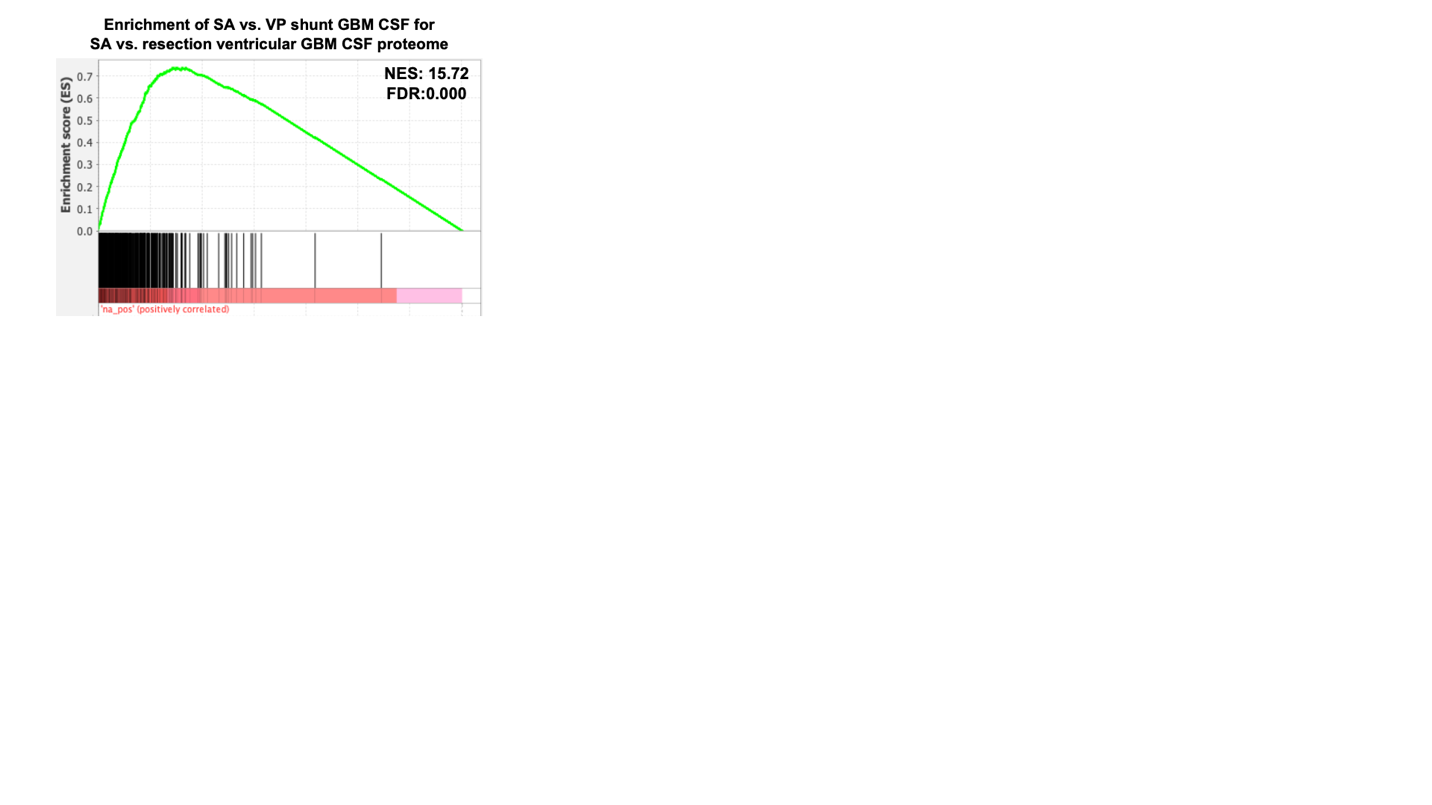
**

Ranked fold-change protein lists were generated for two independent cohorts: 1) four ventricular CSF GBM samples from ventriculoperitoneal shunts versus four subarachnoid CSF GBM samples, and 2) 18 ventricular CSF GBM samples from resections versus 17 subarachnoid CSF GBM samples. Enrichment analysis demonstrated significant enrichment of the subarachnoid vs. ventricular CSF signature derived from shunts to that of the one derived from resections.

**
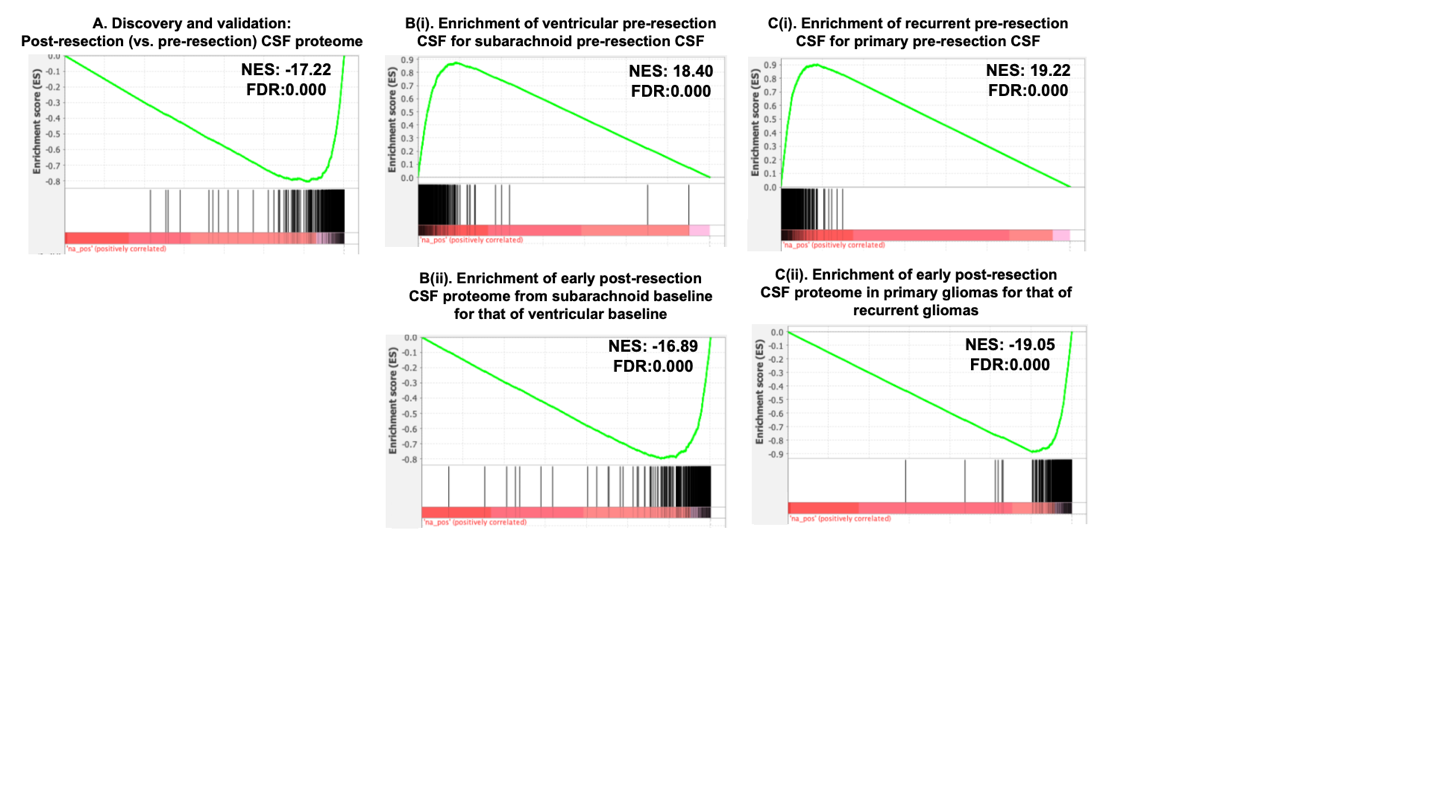
Supplementary Figure 2. Enrichment analyses with paired pre-versus-early post-resection CSF samples from patients with gliomas.**

**
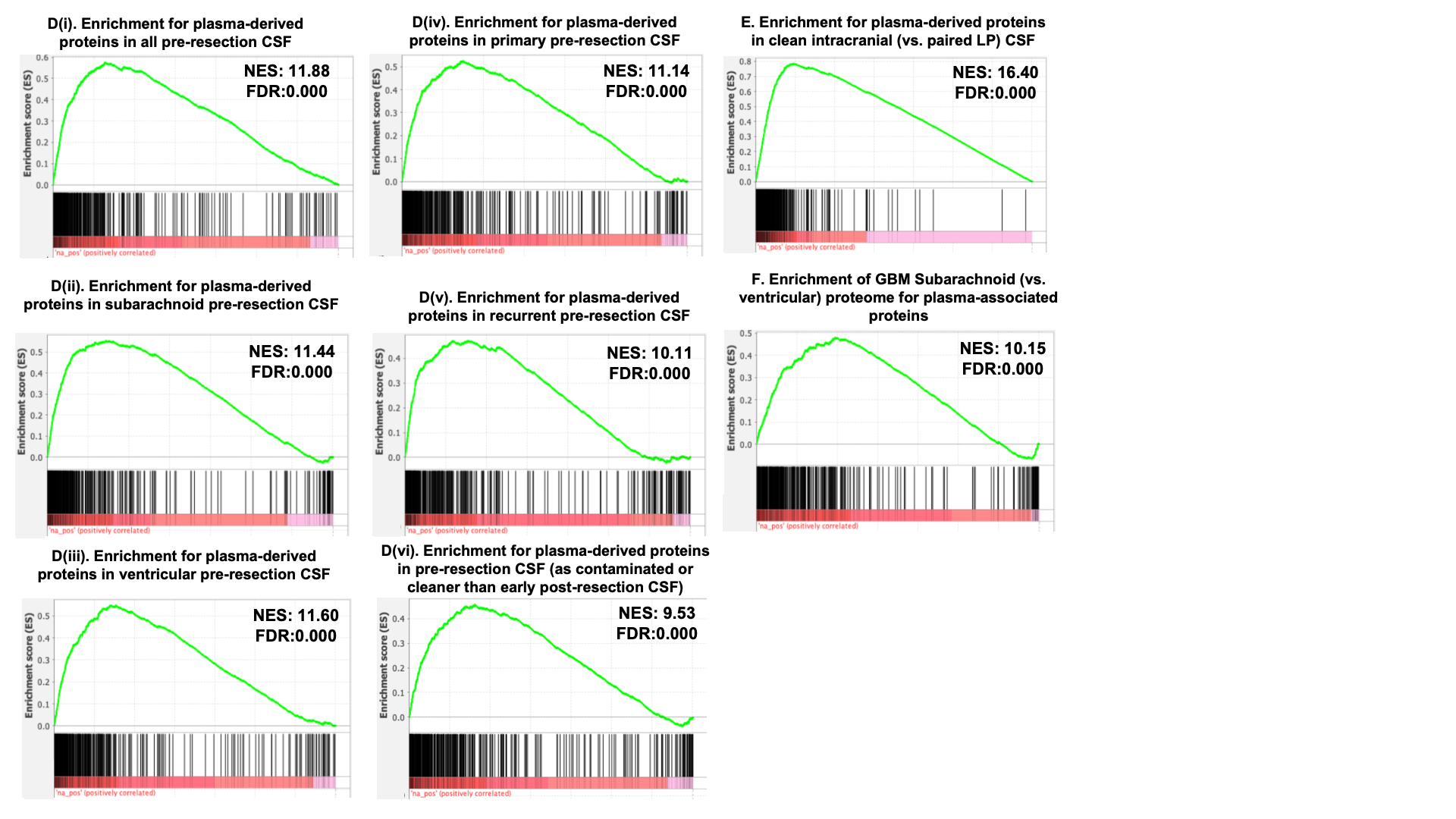
**

**A.** Enrichment of the discovery cohort’s pre-versus-early post resection CSF proteome for the validation cohort’s early post-resection CSF proteome, indicating overlap in the early post-resection CSF proteomes across discovery and validation cohorts.

**B**. The 20 paired pre-versus-post-resection CSF samples were split into two cohorts based on whether the pre-resection CSF sample was obtained from the subarachnoid or ventricular space. The ventricular cohort was used for the protein library sets (top 350 proteins from fold-change). The subarachnoid cohort was used for the ranked list (pre-versus-early post-resection). Enrichment analysis was performed, demonstrating **(i)** enrichment of the pre-resection ventricular and subarachnoid CSF proteomes for one another, when each were compared to post-resection CSF and **(ii)** enrichment of the early post-resection CSF proteomes for both the ventricular and subarachnoid cohorts.

**C**. Similar analyses to Supp. Figure 1B were performed, splitting the cohorts based on whether the patient had a primary or recurrent glioma. Enrichment analysis revealed **(i)** enrichment of the pre-resection CSF proteome of primary and recurrent glioma for one another, when each were compared to their respective early post-resection CSF samples, and **(ii)** enrichment of the early post-resection CSF proteomes for both the primary and recurrent cohorts.

**D-F.** Enrichment analysis for plasma-derived proteins, as defined based on paired bloody and clean CSF samples (adjusted p-value<0.1), was evaluated in the **D)** (i) all pre-versus-early post-resection samples ranked protein list (n=20 pairs), and subgroups within that of (ii) subarachnoid pre-resection samples only (n=11 pairs pairs), (iii) ventricular pre-resection samples only (n=7 pairs), (iv) primary pre-resection samples only (n=14 pairs), (v) recurrent pre-resection samples only (n=6 pairs), (vi) pre-resection samples that were cleaner than or just as contaminated as the early post-resection CSF (n=11 pairs), **E)** intracranial versus lumbar puncture ranked protein list for clean intracranial CSF samples (n=6 pairs), and **F)** glioblastoma (GBM) subarachnoid versus ventricular ranked protein list (unpaired; n=23 patients with subarachnoid CSF versus n=20 patients with ventricular CSF).

Significance was set at NES>10 and FDR<0.001 for all enrichment analyses. NES= normalized enrichment score. FDR = false discovery rate.

**Supplementary Figure 3. Enrichment analyses in paired early and delayed post-resection CSF samples from 9 patients with gliomas.**

**
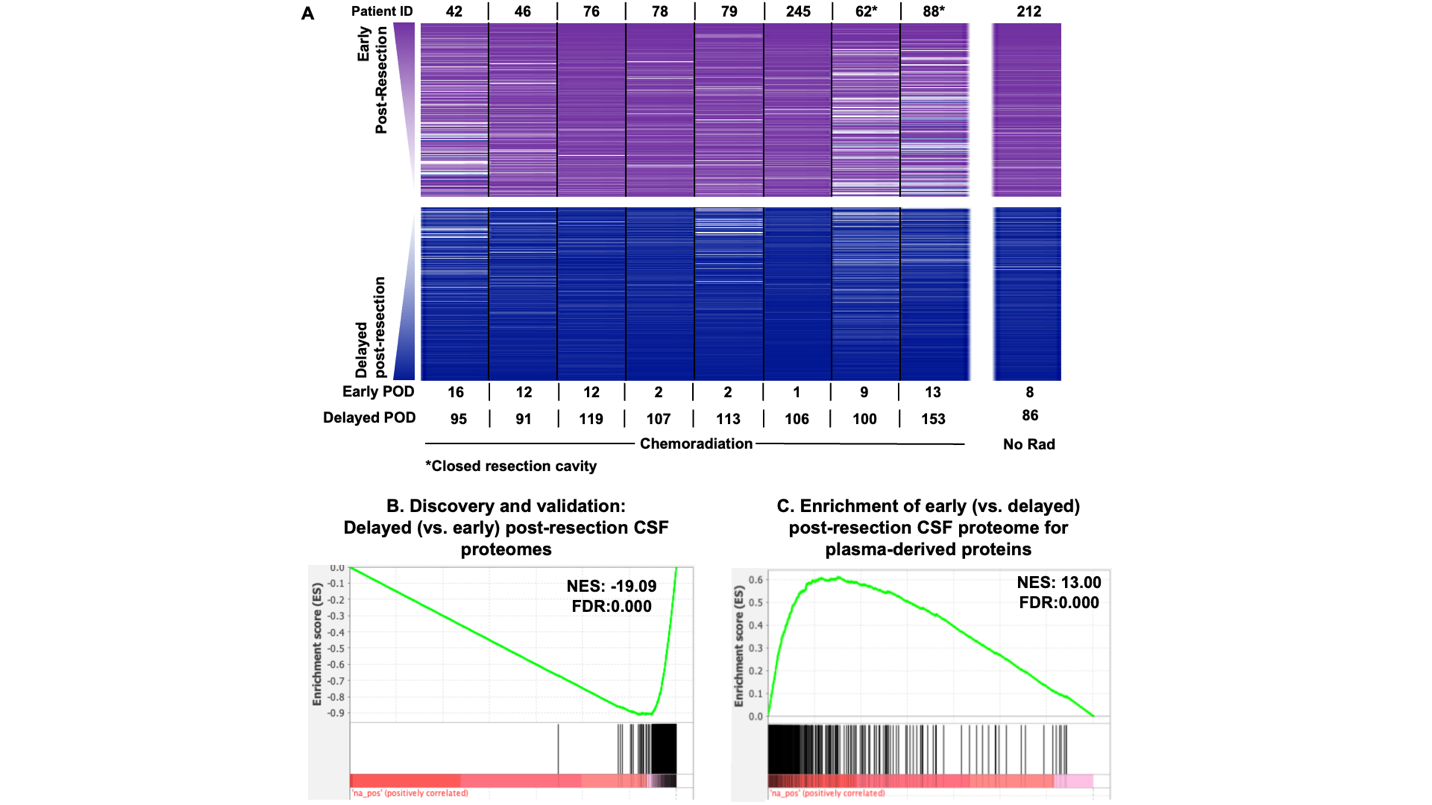
**

**A.** Ranked fold-change lists were generated from each patient’s paired early-versus-delayed post-resection CSF samples (n=9), as in Figure 3Bi. Ranked protein order is shown as a heatmap from 1 (higher early post-resection; purple) to 7,011 (higher delayed post-resection; blue). Eight patients received chemoradiation during this time frame. One patient (#212) did not. The average rank across the 8 patients who received chemoradiation was used to rank all 9 ranked lists. The top and bottom 200 proteins are shown.

**B.** Enrichment in the discovery cohort’s early-versus-delayed post-resection CSF proteome for the validation cohort’s delayed post-resection CSF proteome, indicating overlap in the delayed post-resection CSF proteomes across discovery and validation cohorts.

**C**. Enrichment analysis for plasma-derived proteins, as defined based on paired bloody and clean CSF samples (p-value<0.1) was evaluated in the early-versus-delayed post-resection ranked protein list.

**Supplementary Figure 4. Enrichment analyses in the paired pre-resection versus delayed post-resection CSF samples from 11 patients with gliomas.**


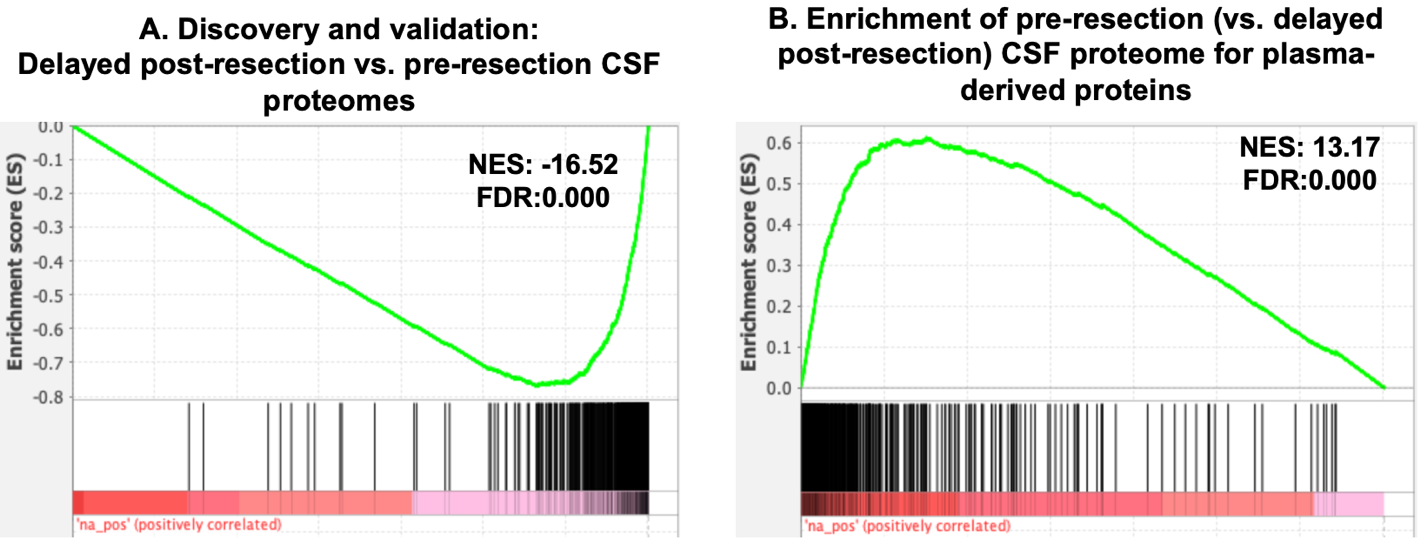


**A.** Enrichment in the discovery cohort’s pre-resection-versus-delayed post-resection CSF proteome for the validation cohort’s delayed post-resection CSF proteome, indicating overlap in the delayed post-resection CSF proteomes across discovery and validation cohorts when compared to the pre-resection CSF proteome.

**B**. Enrichment analysis for plasma-derived proteins, as defined based on paired bloody and clean CSF samples (p-value<0.1), was evaluated in the pre-resection-versus-delayed post-resection ranked protein list.

**Supplementary Figure 5**. **Evaluation of candidate monitoring and pharmacodynamic proteins in longitudinal CSF samples.**

**
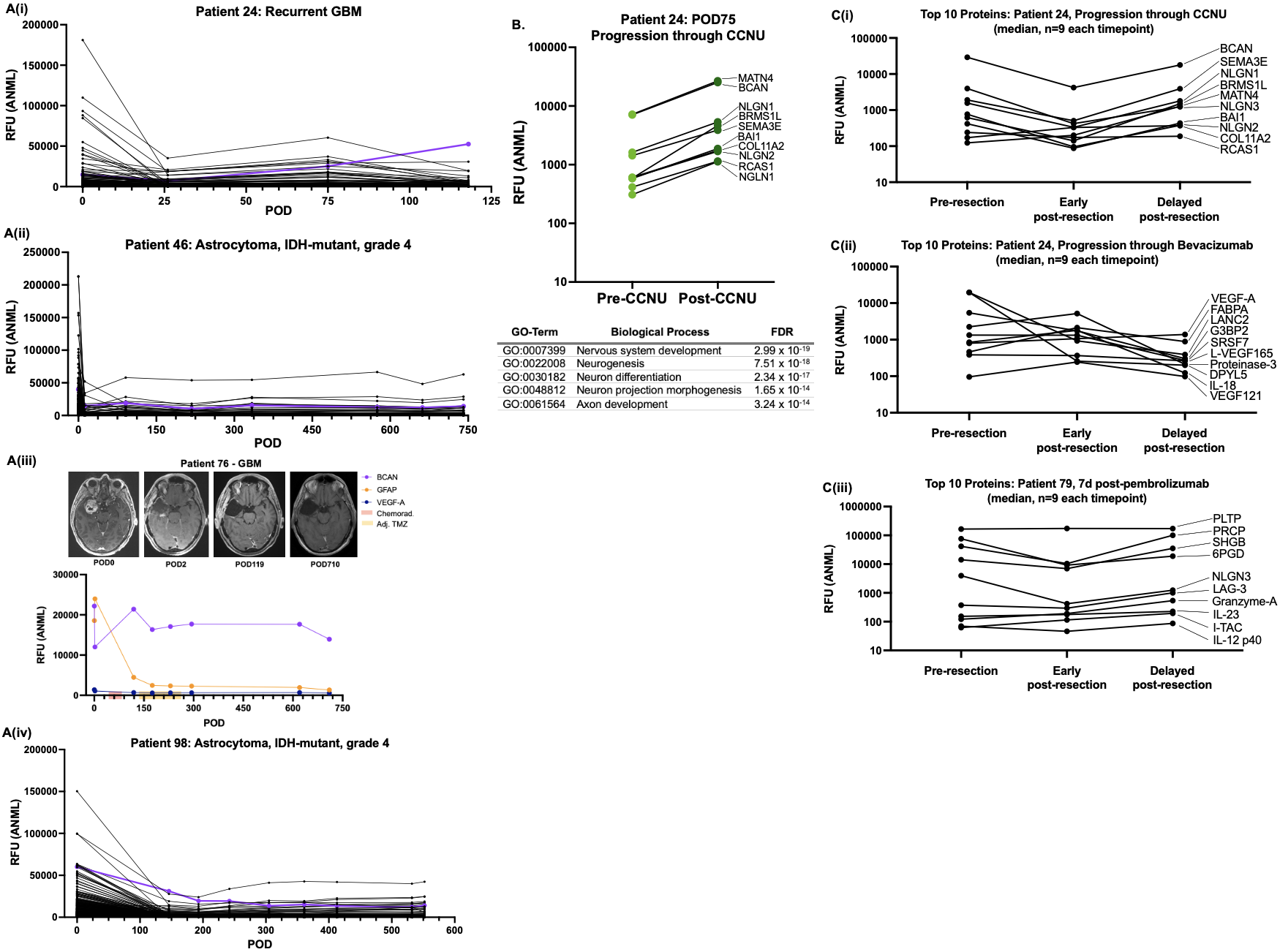
**

**A.** The top 25% of proteins decreasing with resection from **Fig. 2A** were plotted over time starting from resection (post-operative day (POD) 0) in patients **(i)** 24, **(ii)** 46, and **(iv)** 98. Brevican (BCAN) is plotted in purple as the identified protein that best correlated with disease course across all three patients. **(iii)** BCAN, glial fibrillary acidic protein (GFAP), and vascular endothelial growth factor A (VEGF-A) were plotted overtime in a patient with a glioblastoma that remained stable after resection and standard-of-care chemoradiation and adjuvant TMZ.

**B.** Protein fold-changes were calculated in the samples obtained after versus before progression through CCNU (or lomustine) in patient 24. The top 10 proteins based on fold-change were plotted. Protein network enrichment analysis was performed on the top 200 proteins based on fold-change of proteins pre-versus-post-progression through CCNU – five pathways are shown on the table.

**C.** Nine patients had CSF samples obtained at pre-resection, early post-resection, and delayed post-resection timepoints. The median abundance for each of the 10 proteins shown in Supplementary Figure 4B and Figure 4Di-ii are plotted from each timepoint in **(i), (ii),** and **(iii)** respectively. The median is based on 9 patients at each timepoint.

**Supplementary Figure 6. The impacts of anatomy and resection on the CSF proteome were similar in IDH-mutant versus IDH-wild type gliomas.**


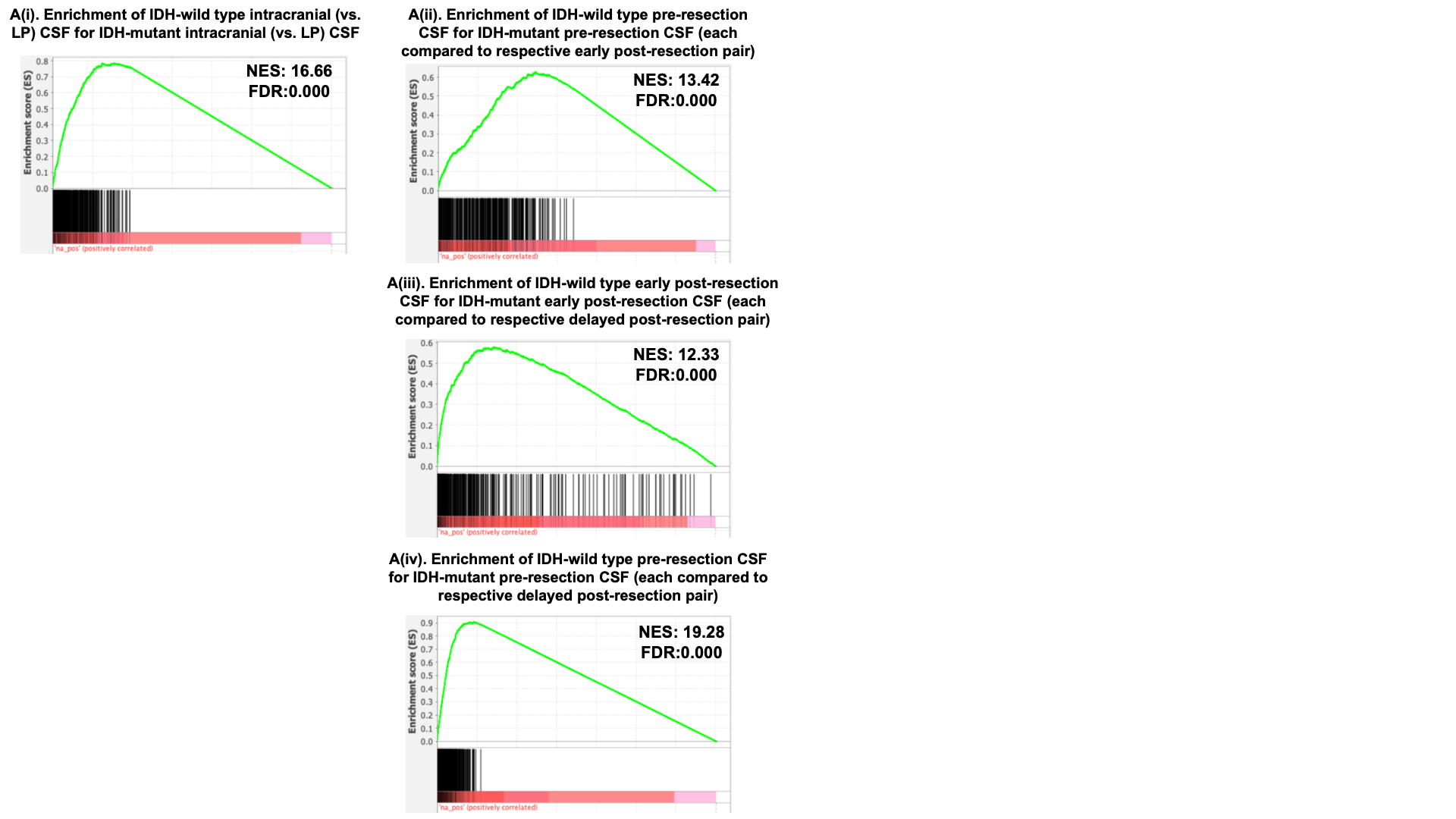


**A(i)** Ranked protein fold-change lists of intracranial vs. lumbar CSF were generated separately for IDH-mutant (n=9 pairs) and IDH-wild type (n=5 pairs) gliomas. Enrichment analyses were then performed, demonstrating significant overlap of the IDH-mutant intracranial vs. lumbar CSF proteome for that of the IDH-wild type intracranial vs. lumbar CSF proteome.

**(ii)** Ranked fold-change lists of pre-versus-early post-resection CSF were generated separately for IDH-mutant (n=10 pairs) and IDH-wild type (n=10 pairs) gliomas. Enrichment analyses demonstrated significant overlap of the IDH-wildtype glioma pre-resection CSF proteome for that of the IDH-mutant glioma pre-resection CSF proteome.

**(iii)** Similar analyses to (ii) were performed for early-versus-delayed resection paired samples (n=4 IDH-mutant; n=5 IDH-wild type gliomas). Enrichment analyses again revealed overlap of the IDH-mutant versus IDH- wild type early-versus-delayed post-resection CSF proteomes.

**(iv)** Similar analyses to (ii-iii) were performed for pre-resection versus delayed post-resection paired samples (n=6 IDH-mutant gliomas; n=5 IDH-wild type gliomas). Significant overlap was once again observed between the IDH-mutant and IDH-wild type groups.

**Supplementary Notes:**

***Interactions of variables***

As noted in the discussion, in non-paired comparisons in the manuscript (subarachnoid vs. ventricular; primary vs. recurrent), some variables which may have an effect on relative protein abundance were not balanced between the groups that we compared. Based on biological reasoning from the results described in the manuscript, the variables *primary versus recurrent* and *male versus female* were hypothesized to interact with the subarachnoid vs. ventricular comparison. Similarly, male vs. female and subarachnoid vs. ventricular imbalances may impact the primary vs. recurrent comparison. To control for these potential interacting variables, we proceeded as follows. For each individual protein we estimated a linear regression model, in which the abundance of that protein is described as a linear function of (1) our variable of interest (e.g. whether a sample was taken pre or post resection), and (2) the potential interacting variables. For a given protein, the coefficient of our variable of interest, thus, reflects the extent of differential enrichment which our model attributes to the variable of interest. To correct for multiple hypothesis testing we, moreover, adjusted the p-values of the coefficients of our variable of interest across the panel of proteins using the Benjamini-Hochberg procedure. The results of this analysis for the comparisons of subarachnoid vs. ventricular (regressing males versus females; primary vs. recurrent) and primary versus recurrent (regressing subarachnoid vs. ventricular; males vs. females) can be found in Supplementary Tables 13 and 14.

***IDH mutation***

All analyses in the main manuscript grouped together IDH-mutant and IDH-wild type gliomas. To determine whether results were reproducible when IDH-mutant gliomas were separated from IDH-wild type ones, the following analyses were re-performed according to IDH-mutation status: lumbar vs. intracranial, pre-versus-early-post-resection, early-versus-delayed post-resection, and pre-versus-delayed post-resection CSF. In all enrichment analyses, CSF proteomic signatures were significantly enriched between IDH-mutant and IDH-wild type groups (**Supplementary Figure 6)**, indicating the reproducibility of anatomical location and resection impacts independent of IDH-mutation status.

Additionally, to determine whether any proteomic signature of IDH mutation exists at baseline, we compared ventricular CSF from 6 grade 4 IDH-mutant grade 4 astrocytomas to the 20 ventricular GBMs, matching for tumor grade, subtype (astrocytoma) and CSF location of origin. Prior to multiple hypothesis correction, 146 proteins were significantly more abundant in GBMs and 268 were significantly more abundant in grade 4 IDH-mutant astrocytomas (Fold-change>1.5, p-value<0.05). However, none of these proteins were significant after multiple hypothesis correction **(Supplementary Table 15)**. As such, if proteomic differences exist between IDH-mutant and IDH-wild type gliomas, they likely have a small effect size that will require a larger sample size to detect more reliably.

**Supplementary References**

**1.** Rohloff JC, Gelinas AD, Jarvis TC, et al. Nucleic Acid Ligands With Protein-like Side Chains: Modified Aptamers and Their Use as Diagnostic and Therapeutic Agents. *Molecular Therapy - Nucleic Acids.* 2014; 3:e201.

**2.** Timsina J, Gomez-Fonseca D, Wang L, et al. Comparative Analysis of Alzheimer's Disease Cerebrospinal Fluid Biomarkers Measurement by Multiplex SOMAscan Platform and Immunoassay-Based Approach. *J Alzheimers Dis.* 2022; 89(1):193-207.

**3.** Gold L, Ayers D, Bertino J, et al. Aptamer-based multiplexed proteomic technology for biomarker discovery. *PLoS One.* 2010; 5(12):e15004.
